# Simulation-Based Mastery Learning with Deliberate Practice Improves Clinical Performance in Spinal Anesthesia

**DOI:** 10.1155/2014/659160

**Published:** 2014-07-16

**Authors:** Ankeet D. Udani, Alex Macario, Kiruthiga Nandagopal, Maria A. Tanaka, Pedro P. Tanaka

**Affiliations:** ^1^Department of Anesthesiology, Perioperative and Pain Medicine, 300 Pasteur Drive, Room H3580, Stanford University, Stanford, CA 94305-5640, USA; ^2^Department of Anesthesiology, Perioperative and Pain Medicine, and Department of Health Research and Policy, Stanford University, Stanford, CA, USA; ^3^Stanford Center for Medical Education Research and Innovation, Stanford University, Stanford, CA, USA

## Abstract

*Introduction.* Properly performing a subarachnoid block (SAB) is a competency expected of anesthesiology residents. We aimed to determine if adding simulation-based deliberate practice to a base curriculum improved performance of a SAB.* Methods.* 21 anesthesia residents were enrolled. After baseline assessment of SAB on a task-trainer, all residents participated in a base curriculum. Residents were then randomized so that half received additional deliberate practice including repetition and expert-guided, real-time feedback. All residents were then retested for technique. SABs on all residents' next three patients were evaluated in the operating room (OR).* Results.* Before completing the base curriculum, the control group completed 81% of a 16-item performance checklist on the task-trainer and this increased to 91% after finishing the base curriculum (*P* < 0.02). The intervention group also increased the percentage of checklist tasks properly completed from 73% to 98%, which was a greater increase than observed in the control group (*P* < 0.03). The OR time required to perform SAB was not different between groups.* Conclusions.* The base curriculum significantly improved resident SAB performance. Deliberate practice training added a significant, independent, incremental benefit. The clinical impact of the deliberate practice intervention in the OR on patient care is unclear.

## 1. Introduction

Research in expert performance identifies deliberate practice as the hallmark of superior performance. Deliberate practice training as described by Ericsson and colleagues entails (1) motivated learners, (2) well-defined learning objectives, (3) precise measurements of performance, (4) focused and repetitive practice, and (5) informative real-time feedback concerning performance [1]. Deliberate practice has been shown to be effective in increasing performance skills in various domains including music, sports, and games such as chess and typing [2, 3]. Recently, educators in science and medicine have been using principles of deliberate practice to design training modules in an attempt to improve student performance [4]. Simulation technologies in particular have been used in the deliberate practice of procedural skills at the graduate medical education level as there is opportunity for repeated practice and immediate feedback in controlled, safe, representative scenarios.

Simulation-based instruction of procedural skills in medicine is becoming widespread. Simulation-based medical education has been shown to increase knowledge, provide opportunities for practice, and allow for assessment [4, 5]. Despite these benefits, the methodology used in simulation variesby instructor, institution, and available resources. Rigorous evaluation of educational techniques such as simulation requires standardized protocols, which, to date, are lacking [6]. Deliberate practice training in simulation-based instruction has been shown to be effective in promoting learning and retention in the performance of lumbar punctures and central line placement [7, 8]. However using deliberate practice to train residents to perform subarachnoid blocks, an expected competency [9], has not been studied, especially to determine whether it can actually change clinical performance on real patients. The most common method for learning this fundamental skill is through apprenticeship with a faculty anesthesiologist. Additional instructional methods include viewing online videos and tutorials, textbooks, workshops, lectures, and simulation-based training [10]. The efficacy of these various educational techniques to achieve competency in the technical performance of a subarachnoid block is unknown.

More generally, the assessment of procedural skills in anesthesiology can be improved compared with other domains of learning and has fallen behind other fields [11]. Thus, the goals of our study were to (1) use a Delphi method to develop the recommended sequence of steps for placement of a subarachnoid block, (2) use this procedural checklist to create a base standardized curriculum consisting of written material and a teaching video, (3) determine whether this base curriculum compared with the base curriculum plus mastery learning through deliberate practice could improve the technical performance of a subarachnoid block on a task-trainer simulator, and (4) determine whether clinical performance of this procedure on patients having joint replacement surgery was improved by either curriculum or both curricula. The primary outcomes were percentage of checklist tasks performed correctly. We also measured the operating room time used to place a subarachnoid block in actual patients.

## 2. Methods

### 2.1. Checklist Development

A checklist of the necessary procedural steps for block placement was adapted from previous neuraxial block checklists [12–14]. Then, a modified Delphi-approach was used to refine and ensure face and content validity. This method is designed to achieve consensus among experts assembled to serve as a panel [15, 16]. Each action was listed in order and given equal weight using a dichotomous scoring system (“satisfactory” or “unsatisfactory”). The initial checklist was designed by 1 author, pilot-tested on a group of 3 local faculties, and then reviewed by 5 board-certified anesthesiologists from four different hospitals to answer specific questions and give feedback. Suggestions for adding or deleting steps were encouraged, and the checklist was reviewed iteratively by the panel until consensus was achieved.

Written teaching materials including the procedural checklist, FAQs, and technique description were produced and modified using the same Delphi-approach described above. A 15-minute video was also produced that provided step-by-step instructions corresponding to the procedural checklist.

**Figure 1:**
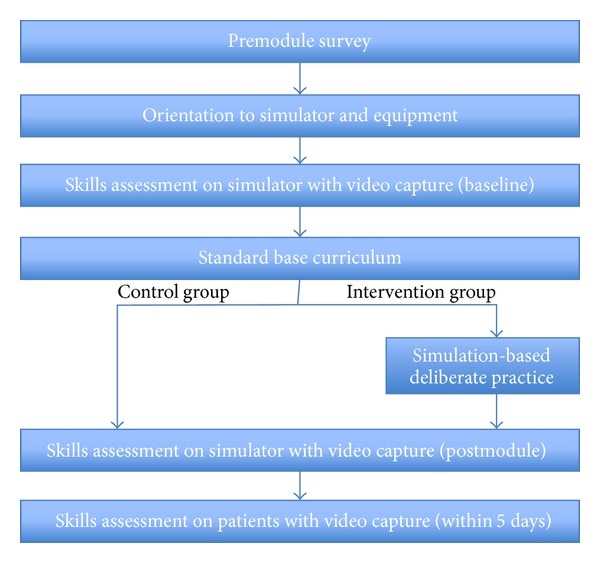
Study flow chart following informed consent and enrollment.

The performance assessment parts of the study were conducted in several phases (Figure 1). The IRB determined this study to be exempt. Stanford anesthesiology PGY2 residents were recruited to participate in the study. Each resident completed a survey to collect demographic data; prior experience with spinal and epidural anesthetics and lumbar punctures, prior practice on a subarachnoid or epidural block task-trainer, and subjective comfort level in performing spinal anesthesia (5-point ordinal scale) were obtained via survey.

### 2.2. Task-Trainer Performance Assessment

A baseline assessment of each participant performing a subarachnoid block was made on a task-trainer (Lumbar Puncture Simulator II, Kyoto Kagaku, Japan) before they were exposed to the base curriculum. The video-recorded performances at baseline were later scored by two authors (A. D. Udani and P. P. Tanaka), one of whom was blinded to which group the participant was in. The assessments used the 16-item checklist developed in the Delphi process. Each item was graded as either satisfactory or unsatisfactory by two trained faculty raters. After the control group residents finished the base curriculum, they received no further training and underwent immediate testing via a second skills assessment on the same task-trainer, on the same day. This was also videotaped and scored in the same fashion.

It was assumed that before exposure to the base curriculum residents would properly complete 65% of the tasks properly in correct order and power calculation (*n* = 9 for each group) indicated that an increase to 95% after the deliberate practice curriculum could be detected with alpha = 0.05 and beta = 0.6.

### 2.3. Clinical Performance Assessment

One to 5 days after completion of the base curriculum residents were videotaped performing subarachnoid blocks in the operating room on 3 consenting patients. These were the first three patient blocks placed by the resident participant since completing their educational module. The same two faculty raters using the same 16-item checklist scored their performance and recorded the time to achieve subarachnoid block. Time was measured using a stopwatch for three contiguous intervals: patient positioning (from patient in room to sitting position), setup (from sitting position to injection of local anesthetic wheal), and subarachnoid injection (from injection of local anesthetic wheal to completion of subarachnoid injection).

Power analysis showed that a sample size of 9 patients in each group would provide sufficient power (alpha 0.05 and beta 0.6) to detect a 3-minute decrease in time from positioning to injection if the baseline time equaled 9 minutes (SD 4 mins) for these junior residents. The 9 minutes was based on prestudy data collected on 6 residents.

Residents were also randomized via a random number generator such that, in addition to the base curriculum, half received simulation-based deliberate practice under the guidance of one faculty anesthesiologist (P. P. Tanaka).

### 2.4. The Mastery Learning with Deliberate Practice Model of SAB

Fundamental principles of deliberate practice training were followed, including (1) having motivated learners (residents volunteered to improve specific aspects of their performance), (2) giving well-defined learning objectives (goals were broken down into specific steps), (3) providing precise measurements of performance (steps corresponded to specific actions), (4) engaging in focused and repetitive practice (residents engaged in specific activities and steps performed unsatisfactorily were repeated until performed satisfactorily), and (5) giving informative real-time feedback concerning performance (residents received one-on-one faculty coaching).

### 2.5. Statistical Analysis

Proportions of participants' gender and exposure to spinal simulator prior to the study were compared using the Fisher exact test. Number of neuraxial blocks prior to the study and perceptions of comfort performing spinal anesthesia were compared among the 2 groups using the nonparametric Mann-Whitney* U* test (http://www.socscistatistics.com/). The Cohen kappa coefficient was used to assess interrater reliability. To assess the impact of the added deliberate practice training, baseline and posttest checklist scores were compared using analysis of covariance to determine if the scores were more improved for the intervention group than for the control group.

## 3. Results

The modified Delphi method resulted in a 16-item checklist of required procedural tasks (Table 1). We used this procedural checklist to create a base curriculum of teaching materials (Appendices A and B) and a 15-minute video (available at http://www.youtube.com/watch?v=eblMcptvcAo&feature=youtu.be).

All 21 residents invited to be in the study consented. The control group had more experience as anesthesia residents, self-reported experience with epidural blocks and simulation training, and higher self-rated comfort performing the spinal anesthesia procedure (Table 2). Scoring of the videos of the residents performing the subarachnoid blocks demonstrated very good agreement (kappa = 0.938 SE = 0.044, 95% confidence interval: 0.852 to 1.0) between examiners.

Before completing the base curriculum, the control group properly completed 81% (SD = 7%, median 81%, and range 69–94%) of the 16 checklist tasks on the task-trainer simulator and this significantly increased to 91% (SD = 7%, median 94%, and range 81–100%) after finishing the base curriculum (*P* < 0.02).

The intervention group (the base curriculum plus deliberate practice) also significantly increased the percentage of checklist tasks properly completed on the task-trainer from 73% (SD = 15%, median 75%, and range 31–88%) to 98% (SD = 4%, median 100%, and range 88–100%), which was a significantly greater increase than observed in the control group (*P* < 0.03).

We were unable to study a full set of 3 patients having subarachnoid block placed per resident due to resident unavailability. In an average of 3.22 (SD 1.2, median 3, and range 2–5 days) days after finishing the curriculum, the control group (*n* = 10 residents) successfully performed spinals on 20 of 21 patients (66% female, mean age 66 SD 12, mean BMI 28 SD 4.45, and range 19–34), with 1 of the spinals ultimately done by the attending (supervising) physician after the resident had prolonged difficulty. The intervention group (*n* = 11) successfully performed spinals on 21 of 28 patients (50% female, mean age 61 yrs SD9, BMI 27 SD 4.9, and range 19–42), as 7 were placed by the attending after the resident had prolonged difficulty.

The control group properly performed 84% (SD = 7%, median 88%, and range 69–94%) of checklist tasks versus 81% (SD = 14%, median 81%, and range 50–100%) of the intervention group (*P* = NS).

The control group on average spent 296 seconds (SD = 104, median 283, and range 113–464) from patient in room to sitting position and 252 seconds (SD = 118, median 260, and range 46–474) from sitting position to injection of local anesthetic wheel while the intervention group (the base curriculum plus deliberate practice) on average spent 253 seconds (SD = 91, median 226, and range 125–517) from patient in room to sitting position and 338 seconds (SD = 91, median 338, and range 158–521) from sitting to injection (in each case, *P* = NS).

Excluding the cases where the attending finished the spinal, time from injection of local anesthetic wheel to finish of subarachnoid injection was also not different for the two groups. This time equaled 253 seconds (SD = 156, median 201, and range 88–627) for the control group and 232 seconds (SD = 158, median 142, and range 86–527) for the intervention group (*P* = NS).

## 4. Discussion

The base curriculum we developed for teaching subarachnoid blocks significantly increased correct performance of the technical aspects of the block in the control group. Importantly, the addition of deliberate practice led to a significantly higher increase in performance in the intervention group. As the depth and breadth of anesthesiology grow and as house staff time and resources are limited, it is important to determine which teaching methods yield the greatest learning [17]. In the current study, we used a rigorous procedure (the Delphi method) to establish a base curriculum and also examined the additional benefits of 1 : 1 mentoring according to predetermined guidelines of deliberate practice.

Although overall benefits persisted several days later on actual patients, no differences in time required to place the blocks or checklist scores were observed between groups. This can be attributed to differences in learning climate between the simulated and operating room environments. Learning climate is defined as the tone or atmosphere of the teaching setting. Some key components of the learning climate may have impacted performance of residents. The operating room may be a challenging educational environment. There is noise from surgical instruments being set up, production pressure from the surgical team, and inherent patient characteristics may complicate subarachnoid block placement. An influential factor may be specific teaching behaviors by attending anesthesia faculty. For example, the time required for the subarachnoid injection was the most difficult of the three periods measured because attendings decided based on their own judgment when to intervene. Instead of encouraging residents to take a different approach to perform the SAB, we observed that the attendings would take over the procedure after a short period of time. The study protocol set no criteria a priori on how the attending should assist the resident and if and when the attending could intervene. The differences in learning climate between the simulated and operating room environments hindered consistency most ideal for resident education and research.

The current study yielded several informative products and results. First, we used a rigorous, iterative methodology to establish a standard base curriculum. This curriculum is currently available to all residents to access via the Internet at any time. Second, we developed a training module using the base curriculum that significantly improved block placement performance. Third, we implemented a deliberate practice training component, which yielded additional performance benefits. Although we were unable to detect a difference in clinical performance between groups trained with the base curriculum and those with the additional deliberate practice, the gaps identified via the current study are being used to design future structured training. For example, we will emphasize the importance of the safety time-out and washing hands before wearing sterile gloves. Residency programs can also use our checklist evaluation to identify deficiencies in trainees and those who require extra instruction. Furthermore, our observations regarding potential confounding variables when transferring to clinical settings can inform future training initiatives.

Simulation-based medical education translational studies attempt to demonstrate that results achieved in the educational laboratory (T1) transfer to improved downstream patient care practices (T2) and improved patient and public health (T3) [18]. Designing and implementing a T2 study comes with inherent difficulties. In fact a recent literature review of hundreds of simulation studies found that only 10% included follow-up data from the clinical environment [19]. It is therefore perhaps not surprising that although we obtained a positive T1 result (improved performance in a simulated subarachnoid block for both groups, particularly the deliberate practice group), this did not translate into a positive T2 result.

This study has several limitations. First, the study enrolled a relatively small group of residents, 21 (although this figure represented 88% of the 24 residents in the PGY2 class). This highlights the challenge of single institution graduate medical education studies as there is usually too few available house staff to enroll to get larger sample sizes. Also, the residents enrolled in the study were at varying points in training and on average were approximately half way through PGY2 year. This variation led to differences in resident neuraxial block placement and simulator experience prior to study enrollment. It is likely that the impact of the teaching residents received on performance improvement is greatest for beginner residents in their first months of residency. The difference between the control and intervention group's training prior to enrollment may also explain the difference in baseline SAB skills, 81% versus 73%, respectively. The impact of starting at a lower baseline score may overstate the overall change described from deliberate practice in the intervention group. However, the control group's prior experience with the spinal simulator may also have influenced the higher baseline performance score. Finally, attending physicians are more likely to take over procedures from junior residents, those with little training experience. This may have resulted in 7 incomplete blocks in the intervention group versus 1 in the control group. Multi-institution studies may be a way to enroll subjects more quickly and increase sample size, although it may be even more difficult to control for prior experience and skills. Another limitation is that posttesting occurred immediately after training potentially enhancing recall.

## 5. Conclusions

The current study represents an advance in simulation-based education, particularly in anesthesia, due to the development and implementation of a base curriculum and the formalized methodology of deliberate practice training. Moreover, this study is one of few investigations examining transfer to clinical settings. Our results and observations have identified specific considerations and areas for improvement in subsequent training modules. For example, this study focused on the technical skill required to place a subarachnoid block but there is more to this in actual practice, including the decision of whether a block is indicated and obtaining consent and postoperative follow-up. The best way to teach all of those elements together deserves further study. More generally, this study provides an attempt at rigorous methodology for designing, implementing, and evaluating simulation-based learning interventions in medicine.

## Figures and Tables

**Figure 2:**
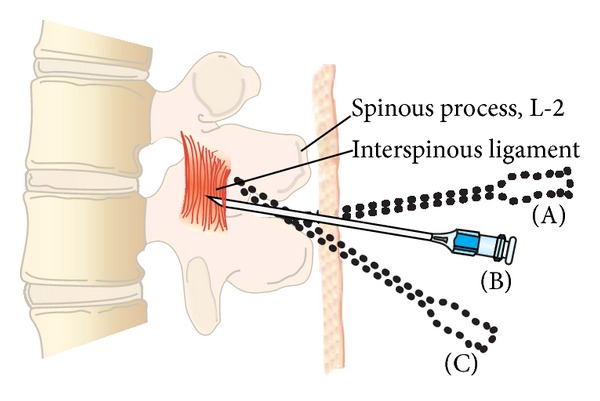
Midline approach to the subarachnoid space. The spinal needle is inserted with a slight cephalad angulation and should advance in the midline without contacting bone (B). If bone is contacted, it may be either the caudad (A) or the cephalad spinous process (C). The needle should be redirected slightly with cephalad and reinserted. If bone is encountered at a shallower depth, the needle is likely walking up the cephalad spinous process. If bone is encountered at a deeper depth, the needle is likely walking down the inferior spinous process. If bone is repeatedly contacted at the same depth, the needle is likely off the midline and walking along the lamina.

**Table 1:** Procedural checklist for subarachnoid block.

Task	Satisfactory	Unsatisfactory
(1) Performs a “time-out” and places monitors on patient (pulse oximetry and NIBP).	—	—

(2) Verifies that spinal kit tray, nonsterile and sterile gloves (correct size), and cleansing solution are present.	—	—

(3) Palpates the superior aspects of the iliac crests and identifies the intersection at the L4 spinous process with nonsterile gloves on. Marks position at the L3/L4 or L4/L5 interspace.	—	—

(4) Cleans the overlying skin with chlorhexidine.	—	—

(5) Opens the spinal tray before placing sterile gloves on.	—	—

(6) Puts on sterile gloves with proper technique.	—	—

(7) Applies sterile drapes.	—	—

(8) Draws up lidocaine in the 3cc syringe and bupivacaine in the 5cc syringe. Administers local anesthesia in a wheal at the previously marked site.	—	—

(9) Injects more anesthetic in the correct location and angle.	—	—

(10) Inserts the introducer needle in the middle of the interspace with a slight cephalad angulation of 10 to 15 degrees. The bevel of the spinal needle should be in the sagittal plane.	—	—

(11) Advances spinal needle through anatomic structures until the subarachnoid space is reached. May experience a popping sensation as the ligamentum flavum is crossed.	—	—

(12) Withdraws the stylet each time a pop is felt to assess for CSF flow.	—	—

(13) Confirms CSF flow by aspiration before and after injecting anesthetic.	—	—

(14) Removes the spinal and introducer needle together once completed.	—	—

(15) Applies pressure with the provided 2 × 2 gauze and assesses good hemostasis.	—	—

(16) Removes the drape, lays the patient, and observes vitals. Disposes of all sharps and biohazard material appropriately.	—	—

**Table 2:** Characteristics of residents enrolled in study (mean (SD, median, and range)).

	Intervention group (deliberate practice)	Control group	*P*value
*N*	11	10	
Months of anesthesia residency completed	5.2 (3.8, 5, 0.25–10)	8.5 (2, 10, 5–12)	0.05
Age (yrs)	28.5 (1.4, 28, 26–31)	30.8 (3.8, 30, 26–37)	0.22
Gender (% F)	45%	20%	0.36
Self-reported number of spinals done before study	6.1 (5, 4, 0–15)	20.0 (16, 14, 3–50)	0.02
Self-reported number of epidurals done before study	9.8 (13, 2, 0–40)	34.5 (16, 30, 15–60)	0.002
Self-reported number of lumbar punctures done before study	5.2 (3, 5, 2–12)	6.5 (5, 7, 0–15)	0.69
Have you practiced on spinal simulator before study (% yes)	55%	0%	0.01
How comfortable are you performing spinal anesthesia (1 = not comfortable, 5 = very comfortable)	2.8 (0.9, 3, 1–4)	3.7 (0.82, 4, 2–5)	0.04

## Appendices

### A. Spinal Anesthesia Teaching Module: FAQs about Spinal Anesthesia

#### A.1. What Agent Should Be Used to Cleanse the Skin for Spinal Anesthesia?

The skin is prepared with an appropriate antiseptic solution and draped. All antiseptic solutions are neurotoxic, and care must be taken not to contaminate spinal needles or local anesthetics with the antiseptic solution. Chlorhexidine-alcohol antiseptic prevents colonization of percutaneous catheters better than does 10% povidone-iodine. Consequently, the American Society of Regional Anesthesia currently recommends chlorhexidine for skin antisepsis prior to regional anesthesia procedures. How one drapes is a matter of personal preference, but clear plastic drapes offer the important advantage of permitting visualization of the entire back, which makes it easier to identify a rotated or inadequately flexed spine.

#### A.2. Should a Mask, Hat, Handwashing, and Gown Be Used in addition to Sterile Gloves?

Full sterile precautions are required for spinal anesthesia. Thorough handwashing greatly reduces the risk of cross-contamination and should occur prior to performing any regional anesthetic technique. Alcohol-based antiseptic solutions will provide the maximal degree of antimicrobial activity with extended duration when compared to nonalcoholic antimicrobial or nonantimicrobial preparations. The duration and method of washing (standard handwashing versus full surgical scrub) required to reduce infectious complications are currently unknown (grade A). Higher microbial counts have been identified in health-care workers who do not remove jewelry prior to handwashing. Therefore, it may be prudent to remove all jewelry items (rings, watches, etc.) prior to handwashing to reduce the risk of contamination. Sterile surgical gloves should be used and considered a supplement to, not replacement for, handwashing (grade B). The use of surgical gloves is advocated not only to protect patients from cross-contamination but also to protect health-care workers from blood-borne pathogen exposure as required by the Occupational Safety and Health Administration (OSHA). The use of surgical masks during regional anesthesia will maximize sterile barrier precautions (grade A). In particular, surgical masks have been found to significantly reduce the likelihood of site contamination from microorganisms grown in the upper airway of clinicians. Although the routine use of masks has not been found to reduce infectious complications related to regional anesthesia, they do remain a vital protective measure against blood-borne pathogen exposure as recommended by the Occupational Safety and Health Administration (OSHA) (grade B).

#### A.3. What Type of Needle Is “Best” to Perform a Spinal Anesthesia?

Spinal needles are classified by the design of their tips. The Whitacre, Eldor, Marx, and Sprotte spinal needles have a “pencil-point” tip with one or two (Eldor) apertures on the side of the shaft proximal to the tip. The Greene, Atraucan, and Quincke needles have beveled tips with cutting edges. The pencil-point needles require more force to insert than the bevel-tip needles but provide a better tactile “feel” of the various tissues encountered as the needle is inserted. In addition, the bevel has been shown to cause the needle to be deflected from the intended path as it passes through tissues while the pencil-point needles are not deflected. Larger gauge (i.e., smaller diameter) spinal needles are less likely to cause postdural puncture headaches (PDPH) but are more readily deflected than smaller gauge needles. Spinal needles are typically sized 22 to 29 gauge. Spinal needles smaller than 22 gauge are often easier to insert if an introducer needle is used. The introducer is inserted into the interspinous ligament in the intended direction of the spinal needle and the spinal needle is then inserted through the shaft of the introducer. The introducer prevents the spinal needle from being deflected or bent as it passes through the interspinous ligament. Needles of the same outside diameter may have different inside diameters. This is important because inside diameter determines how large a catheter can be inserted through the needle and determines how rapidly CSF will appear at the needle hub during spinal needle insertion. All spinal needles come with a tight-fitting stylet. The stylet prevents the needle from being plugged with skin or fat and, importantly, prevents dragging skin into the epidural or subarachnoid spaces, where the skin may grow and form dermoid tumors.

#### A.4. What Other Measures May Reduce PDPH?

Orientating the bevel of the standard spinal needle parallel versus perpendicular to the fibers of the cauda equina has been shown to reduce the risk of postprocedure headache. The reason is not well understood. Bed rest postspinal anesthesia does not reduce the incidence of postdural puncture headache. See [20].

#### A.5. Is There an Advantage to a Sitting versus Lateral Recumbent Position in Performing Spinal Anesthesia?

Careful attention to patient positioning is critical to successful spinal block. Poor positioning can turn an otherwise easy spinal anesthetic into a challenge for both the anesthesiologist and the patient. Studies of the intervertebral distance show that the space is increased slightly (by about 0.2 cm) when the patient is in the sitting position, with the feet supported, compared to the lateral recumbent position. There are no studies comparing “success rates,” ease of puncture, need for second attempts, or other clinical outcomes with the sitting versus recumbent position.

#### A.6. What If My Patient Is Febrile, Can I Place a Spinal Block?

Serious central neuraxial infections such as arachnoiditis, meningitis, and abscess following spinal or epidural anesthesia are rare. Available data suggest that patients with evidence of systemic infection may safely undergo spinal anesthesia, provided appropriate antibiotic therapy is initiated prior to dural puncture, and the patient has demonstrated a response to therapy, such as a decrease in fever (placement of an indwelling epidural or intrathecal catheter in this group of patients remains controversial) (grade B). Epidural catheters should be removed in the presence of local erythema and/or discharge; there are no convincing data to suggest that concomitant infection at remote sites and the absence of antibiotic therapy are risk factors for infection (grade A). A delay in diagnosis and treatment of major CNS infections of even a few hours may significantly worsen neurologic outcome (grade B).

#### A.7. What Factors Increase the Risk of Spinal Hematoma after Spinal Block?

Bleeding disorders may increase the risk of spinal hematoma related to spinal puncture. Spinal hematoma may in turn result in spinal cord compression and permanent paralysis. Most of the literature on the risks of spinal hematoma after lumbar puncture comes from case reports and case series in patients receiving spinal or epidural anesthesia. Because of the variability in the cases and procedures reported in the literature, many experts refrain from specific recommendations about levels of platelets or INR that are “safe” for lumbar puncture.

Additional risk factors for spinal hematoma and neurologic compromise include patient factors (female sex, increased age, ankylosing spondylitis or spinal stenosis, and renal insufficiency), factors related to technique (traumatic needle/catheter placement), and dosing factors (“high” or “low” dose LMWH, timing of LMWH, and concomitant antiplatelet or anticoagulants). There are some guidelines, described below, for specific situations.

#### A.8. Can Spinal Anesthesia Be Performed Safely in Patients with Thrombocytopenia?

A recent extensive review concluded with the recommendation that LP may be safely performed in patients with a platelet count ≥40 K, as long as the patient is not receiving antiplatelet agents or anticoagulants, there are no other coagulopathies, the platelets are functioning normally, and the platelet count is stable. This is consistent with the recommendations of the American National Red Cross (http://www.redcross.org/www-files/Documents/WorkingWiththeRedCross/practiceguidelinesforbloodtrans.pdf); see [21].

#### A.9. Can Spinal Anesthesia Be Performed Safely in Patients on Antiplatelet Agents (Aspirin and/or Plavix)?

Patients taking nonsteroidal anti-inflammatory drugs with antiplatelet effects (e.g., cyclooxygenase-1 inhibitors) or receiving subcutaneous unfractionated heparin for deep vein thrombosis prophylaxis are not viewed as being at increased risk of spinal hematoma.

In contrast, other classes of antiplatelet drugs, like thienopyridine derivatives (e.g., ticlopidine, clopidogrel) and glycoprotein IIb/IIIa antagonists (e.g., abciximab, eptifibatide, and tirofiban), have a more potent effect on platelet aggregation, and neuraxial block should generally not be performed in patients taking these or similar medications. Further, the consensus statement recommends that ticlopidine be discontinued for 2 weeks and clopidogrel for 1 week before performing central neuraxial blocks. The glycoprotein IIb/IIIa antagonists have a shorter duration of action; thus, it is recommended that abciximab should be discontinued 24 to 48 hours before central neuraxial block, and eptifibatide and tirofiban should be discontinued 4 to 8 hours beforehand; see [22, 23].

#### A.10. Can Spinal Anesthesia Be Performed Safely in Patients Receiving Prophylactic Heparin or Low-Molecular-Weight Heparin?

Low-molecular-weight heparin increases the risk of spinal hematoma sufficiently to warrant a “black box warning” about use of neuraxial (spinal or epidural) anesthesia in patients on LMWH. Practitioners are urged to “consider risk versus benefit” and observe the patient closely for bleeding and signs of neurological impairment if therapy is administered during or immediately following lumbar puncture.

Patients receiving fractionated low-molecular-weight heparin (e.g., enoxaparin, dalteparin, and tinzaparin) are considered to be at increased risk of spinal hematoma. Patients receiving these drugs preoperatively at thromboprophylactic doses should have the drug held for 10 to 12 hours before central neuraxial block. At higher doses, such as those used to treat established deep vein thrombosis, central neuraxial block should be delayed for 24 hours after the last dose. For patients in whom low-molecular-weight heparin is begun after surgery, single-shot central neuraxial blocks are not contraindicated provided that the first low-molecular-weight heparin dose is not administered until 24 hours postoperatively if using a twice-daily dosing regimen and 6 to 8 hours if using a once-daily dosing regimen. If an indwelling central neuraxial catheter is in place, it should not be removed until 10 to 12 hours after the last low-molecular-weight heparin dose, and the subsequent doses should not begin until at least 2 hours after catheter removal.

There is no contraindication to spinal anesthesia in patients receiving prophylactic unfractionated heparin as long as the total 24-hour dose is ≤10,000 units; see [22].

#### A.11. Can Spinal Anesthesia Be Performed Safely in Patients with Elevated INR due to Coumadin Use? Can Spinal Anesthesia Be Performed Safely in Patients with Elevated INR due to Their Underlying Medical Condition (e.g., Liver Disease)?

The American National Red Cross recommends using fresh frozen plasma to treat multiple coagulation deficiencies (such as liver disease) prior to an invasive procedure, including lumbar puncture. The goal is an INR ≤ 1.5. FFP may also be used to correct INR in patients on Coumadin if urgent reversal of anticoagulation is needed. The usual dose to provide 30% of plasma factor concentrate is 10–20 mL/kg. A “unit” of FFP is approximately 250 cc, though the volume may vary. In a 70 kg patient, 3–6 units of FFP is recommended to correct INR sufficiently. Complete normalization of INR is often not possible in patients with liver disease.

Patients with hemophilia or von Willebrand's disease should have factors normalized prior to lumbar puncture; see [24, 25].

### B. Spinal Anesthesia Teaching Module: Spinal Anesthesia Technique Description

#### B.1. Midline Approach

From the midline approach to the subarachnoid space, the skin overlying the desired interspace is infiltrated with a small amount of local anesthetic to prevent pain when inserting the spinal needle. Additional local anesthetic (1 to 2 mL) is then deposited along the intended path of the spinal needle to a depth of 1 to 2 inches. This deeper infiltration provides additional anesthesia for spinal needle insertion and helps identify the correct path for the spinal needle. Infiltrating local anesthetic lateral to the midline is painful and generally unnecessary.

The spinal needle or introducer needle is inserted in the middle of the interspace with a slight cephalad angulation of 10 to 15 degrees (Figure 2). The needle is then advanced, in order, through the subcutaneous tissue, supraspinous ligament, interspinous ligament, ligamentum flavum, epidural space, dura mater, and finally arachnoid mater. The ligaments produce a characteristic “feel” as the needle is advanced through them, and the anesthesiologist should develop the ability to distinguish a needle that is advancing through the high-resistance ligaments from one that is advancing through lower-resistance paraspinous muscle. This will allow early detection and correction of needles that are not advancing in the midline. Penetration of the dura mater often produces a subtle “pop” that is most easily detected with the pencil-point needles. Detection of dural penetration will prevent inserting the needle all the way through the subarachnoid space and contacting the vertebral body. In addition, learning to detect dural penetration will allow one to insert the spinal needle quickly without having to stop every few millimeters and remove the stylet to look for CSF at the needle hub.

Once the needle tip is believed to be in the subarachnoid space, the stylet is removed to see if CSF appears at the needle hub. With small-diameter needles (26 to 29 gauge) this generally requires 5 to 10 seconds. If CSF does not appear, the needle orifice may be obstructed by a nerve root and rotating the needle 90 degrees may result in CSF flow. Alternatively, the needle orifice may not be completely in the subarachnoid space and advancing an additional 1 to 2 mm may result in brisk CSF flow. This is particularly true of pencil-point needles, which have their orifice on the side of the needle shaft proximal to the needle tip. Finally, failure to obtain CSF suggests that the needle orifice is not in the subarachnoid space and the needle should be reinserted.

If bone is encountered during needle insertion, the anesthesiologist must develop a reasoned, systematic approach to redirect the needle. Simply withdrawing the needle and repeatedly reinserting it in different directions are not appropriate. When contacting bone, the depth should be immediately noted and the needle redirected slightly with cephalad. If bone is again encountered at a greater depth, then the needle is most likely walking down the inferior spinous process and it should be redirected more with cephalad until the subarachnoid space is reached. If bone is encountered again at a shallower depth, then the needle is most likely walking up the superior spinous process and it should be redirected more with caudad. If bone is repeatedly encountered at the same depth, then the needle is likely off the midline and walking along the vertebral lamina (Figure 2).

When redirecting a needle it is important to withdraw the tip into the subcutaneous tissue. If the tip remains embedded in one of the vertebral ligaments, attempts at redirecting the needle will simply bend the shaft and will not reliably change needle direction. When using an introducer needle, it also must be withdrawn into the subcutaneous tissue before being redirected. Changes in needle direction should be made in small increments because even small changes in needle angle at the skin may result in fairly large changes in position of the needle tip when it reaches the spinal meninges at a depth of 4 to 6 cm. Care should be exercised when gripping the needle to ensure that it does not bow. Insertion of a curved needle will cause it to veer off course.

If the patient experiences a paresthesia, it is important to determine whether the needle tip has encountered a nerve root in the epidural space or in the subarachnoid space. When the paresthesia occurs, immediately stop advancing the needle, remove the stylet, and look for CSF at the needle hub. The presence of CSF confirms that the needle encountered a cauda equina nerve root in the subarachnoid space and the needle tip is in good position. Given how tightly packed the cauda equina nerve roots are, it is surprising that all spinal punctures do not produce paresthesias. If CSF is not visible at the hub, then the paresthesia may have resulted from contact with a spinal nerve root traversing the epidural space. This is especially true if the paresthesia occurs in the dermatome corresponding to the nerve root that exits the vertebral canal at the same level in which the spinal needle is inserted. In this case the needle has most likely deviated from the midline and should be redirected toward the side opposite the paresthesia. Occasionally, pain experienced when the needle contacts bone may be misinterpreted by the patient as a paresthesia and the anesthesiologist should be alert to this possibility.

Once the needle is correctly inserted into the subarachnoid space, it is fixed in position and the syringe containing local anesthetic is attached. CSF is gently aspirated to confirm that the needle is still in the subarachnoid space and the local anesthetic slowly injected (≤0.5 mL/sec). After completing the injection, a small volume of CSF is again aspirated to confirm that the needle tip remained in the subarachnoid space while the local anesthetic was deposited. This CSF is then reinjected and the needle, syringe, and any introducer were removed together as a unit. If the surgical procedure is to be performed in the supine position, the patient is helped onto his or her back. To prevent excessive cephalad spread of hyperbaric local anesthetic, care should be taken to ensure that the patient's hips are not raised off the bed as they turn.

Once the block is placed, strict attention must be paid to the patient's hemodynamic status with blood pressure and/or heart rate supported as necessary. Block height should also be assessed early by pin prick or temperature sensation. Temperature sensation is tested by wiping the skin with alcohol and may be preferable to pin prick because it is not painful. If, after a few minutes, the block is not rising high enough or is rising too high, the table may be tilted as appropriate to influence further spread of hypobaric or hyperbaric local anesthetics.
